# A Simple Preoperative Scoring System for Risk Stratification of Complicated Appendicitis in Children

**DOI:** 10.3390/children13070913

**Published:** 2026-07-10

**Authors:** Yohei Sanmoto, Masanaga Matsumoto, Kouji Masumoto

**Affiliations:** 1 Department of Pediatric Surgery, University of Tsukuba Hospital, Tsukuba 305-8576, Japan; masa.fb.81@gmail.com (M.M.); kmasu@md.tsukuba.ac.jp (K.M.); 2 Graduate School of Comprehensive Human Sciences, University of Tsukuba, Tsukuba 305-8575, Japan

**Keywords:** acute appendicitis, complicated, pediatrics, prediction, risk stratification

## Abstract

**Highlights:**

**What are the main findings?**

- We developed a simple, additive scoring system for predicting complicated appendicitis in children using a small set of routinely available preoperative variables.
- The scoring system demonstrated good discriminative performance and enabled clinically interpretable risk stratification while emphasizing robustness and bedside applicability using prespecified predictors.

**What are the implications of the main findings?**

- This pragmatic scoring system may support standardized early risk assessment and clinical decision-making in cases of pediatric acute appendicitis.

**Abstract:**

**Background/Objectives**: Preoperative discrimination of complicated appendicitis in children remains challenging but crucial for timely surgical decision-making and perioperative management. We aimed to develop a simple, clinically applicable scoring system for preoperative risk stratification of complicated appendicitis in children. **Methods**: This retrospective single-center study included children aged 5 to <16 years who underwent emergency appendectomy between 2015 and 2024. Complicated appendicitis was defined as gangrene, perforation, or intra-abdominal abscesses on intraoperative or pathological evaluation. Literature-based prespecified predictors included body temperature ≥ 38.0 °C, presence of periappendicular fluid, serum sodium level < 135 mEq/L, white blood cell (WBC) count > 12,000/μL, and C-reactive protein (CRP) level ≥ 3.0 mg/dL. A multivariable logistic regression model using Firth’s penalized likelihood was developed and internally validated using bootstrap resampling. An additive scoring system was derived from the final model. **Results**: Among 301 patients, 102 (33.9%) had complicated appendicitis. Based on the Firth penalized multivariable model, integer point values were assigned in proportion to the regression coefficients. Serum sodium level < 135 mEq/L and CRP level ≥ 3.0 mg/dL assigned 2 points each; body temperature ≥ 38.0 °C, presence of periappendicular free fluid, and WBC count > 12,000/μL assigned 1 point each. The scoring system (range, 0–7) demonstrated good discrimination (area under the receiver operating characteristic curve, 0.880; 95% confidence interval, 0.840–0.920), minimal optimism, and good calibration. Stratification into low-, intermediate-, and high-risk categories showed an increasing prevalence of complicated appendicitis. **Conclusions**: This simple preoperative scoring system enables reliable and clinically interpretable risk stratification for complicated appendicitis in children aged 5 to <16 years and may support early decision-making in routine pediatric surgical practice.

## 1. Introduction

Acute appendicitis is one of the most common abdominal surgical emergencies in children [1]. Among pediatric patients, approximately 30–40% of cases are classified as complicated appendicitis, including gangrenous appendicitis, perforation, or abscess formation [2,3,4], which is associated with a substantially greater burden, including higher rates of postoperative infectious complications, longer operative times, prolonged hospitalization, and increased risk of readmission [3].

Despite its clinical importance, current preoperative diagnostic approaches are not always sufficient to reliably distinguish complicated appendicitis from uncomplicated disease or to directly guide the urgency and intensity of treatment. This challenge may be partly attributable to the fact that clinical presentations in children are often atypical or nonspecific, particularly in younger patients, contributing to diagnostic uncertainty and delays in appropriate treatment [5]. Therefore, an early and reliable risk stratification tool is essential to guide clinical decision-making, facilitating timely surgical intervention for patients at high risk of complicated appendicitis and supporting appropriate perioperative antibiotic management, thereby contributing to improved clinical outcomes [6,7]. In this context, numerous studies have investigated preoperative factors to identify complicated appendicitis in children. Several clinical characteristics, including younger age, prolonged symptom duration, and elevated body temperature, have been associated with complicated appendicitis [8,9]. Similarly, blood-based laboratory abnormalities such as leukocytosis, elevated C-reactive protein (CRP) levels, and hyponatremia have attracted attention as objective and reproducible markers with favorable discriminative performance for identifying complicated cases [7,8,10]. However, most of these findings are derived from single-cohort, data-driven analyses, and clinically applicable standardized criteria for their direct use in preoperative risk stratification have yet to be established. Preoperative imaging has been evaluated for the diagnosis of complicated appendicitis in children. Ultrasonographic features, including increased appendiceal diameter and the presence of periappendicular free fluid, have been reported to be suggestive of complicated disease [11,12]. Nevertheless, imaging parameters alone provide limited discriminatory ability, and relatively high false-negative rates have been reported for the preoperative identification of complicated appendicitis [13].

In recent years, multivariable scoring models that integrate clinical, laboratory, and imaging variables have been proposed and have demonstrated promising diagnostic performance [14,15,16]. However, many existing models depend on cohort-specific variable selection and cut-off determination driven primarily by statistical associations within derivation datasets, which may limit their interpretability and consistent applicability across different clinical settings. Accordingly, based on existing evidence rather than cohort-dependent optimization, the present study aimed to develop a clinically applicable, scoring-based preoperative risk stratification tool for complicated appendicitis in children. This tool was intended to support earlier recognition of complicated disease and contribute to potential therapeutic advantages across diverse clinical environments.

## 2. Materials and Methods

### 2.1. Patients and Data Collection

This retrospective observational study included patients aged 5 to <16 years who underwent emergency appendectomy for acute appendicitis at the Department of Pediatric Surgery, University of Tsukuba Hospital, Japan, between January 2015 and December 2024. The exclusion criteria were (1) age younger than 5 years, (2) concomitant abdominal emergency conditions at presentation, and (3) missing data for key clinical variables.

Clinical data were extracted from electronic medical records and included patient characteristics (sex, age, height, body weight, and body mass index), body temperature at presentation, and signs of peritoneal irritation. Preoperative laboratory parameters included white blood cell (WBC) count, hemoglobin level, platelet count, sodium level, and CRP level. Imaging findings were obtained from preoperative abdominal ultrasonography performed in the emergency department by pediatric surgeons, focusing on the presence of periappendicular free fluid. Intraoperative findings included appendiceal perforation and intra-abdominal abscesses, and histopathological evaluation assessed the presence of gangrenous appendicitis. During the study period, emergency surgery was the standard management strategy for acute appendicitis at our institution.

### 2.2. Definitions of Complicated and Uncomplicated Appendicitis

Complicated appendicitis was defined as appendiceal gangrene, perforation, or intra-abdominal abscess identified intraoperatively or on histopathological examination, in accordance with the 2020 World Society of Emergency Surgery Jerusalem guidelines [17]. Uncomplicated appendicitis was defined as histologically confirmed acute appendicitis without any of these features. The classification of complicated versus uncomplicated appendicitis was based solely on intraoperative and pathological findings and was determined independently of all candidate predictors.

### 2.3. Prediction Model Development

Patients were classified as having complicated or uncomplicated appendicitis according to predefined intraoperative and pathological criteria. Model development was conducted in two sequential phases: (1) development and internal validation of a multivariable prediction model, and (2) derivation of a simplified additive clinical scoring system based on the multivariable model.

### 2.4. Candidate Predictor Selection

To minimize overfitting and avoid the development of a purely data-driven model tailored to the present cohort, candidate predictors were predefined a priori based on prior literature. Variables were selected if they had been independently associated with complicated pediatric (perforated) appendicitis or had demonstrated favorable discriminative performance in previous studies [7,8,9,10,11,12,13,14,18,19,20]. Based on this approach, the following predictors were included body temperature ≥ 38.0 °C at emergency department presentation, presence of periappendicular free fluid on ultrasonography, WBC count > 12,000/μL, serum sodium level < 135 mEq/L, and CRP level ≥ 3 mg/dL.

### 2.5. Multivariable Logistic Regression Modeling

All predefined dichotomized predictors were entered simultaneously into a multivariable logistic regression model, with complicated appendicitis as the dependent variable. Given the presence of small subgroup sizes and the potential risk of complete or quasi-complete separation, the model was fitted using Firth’s penalized likelihood logistic regression, which provides bias-reduced parameter estimates under such conditions [21].

### 2.6. Internal Validation and Calibration of the Multivariable Logistic Regression Model

Internal validation was performed using bootstrap resampling with 1000 iterations. For each bootstrap sample, the penalized multivariable model was refitted and evaluated in bootstrap sample and original dataset. Optimism was calculated as the mean difference between the apparent and test area under the receiver operating characteristic curve (AUC), and an optimism-corrected AUC was subsequently derived. Model calibration was assessed using calibration plots by grouping patients into deciles based on the predicted risk and comparing the mean predicted probabilities with the observed event rates within each decile. The Hosmer–Lemeshow goodness-of-fit test was used as a supplementary assessment of calibration.

### 2.7. Development of the Scoring System

An additive clinical scoring system was derived from the multivariable logistic regression model. The regression coefficients from the dichotomized model were used to assign integer point values to each predictor. The predictor with the smallest absolute coefficient was assigned 1 point, and the remaining predictors were scaled proportionally and rounded to the nearest integer. Individual total scores were calculated as the sum of the assigned points.

### 2.8. Reporting Standards

The reporting of this prediction model followed the Transparent Reporting of a multivariable prediction model for Individual Prognosis Or Diagnosis guidelines [22]. Because this retrospective study included all eligible patients during the predefined study period, no a priori sample size calculation was performed; the study size was determined by the number of eligible patients between January 2015 and December 2024.

### 2.9. Outcome Measures

The primary outcomes of interest were the performance characteristics of the derived scoring system. These included (1) the ability to discriminate between complicated and uncomplicated appendicitis and (2) score-based risk stratification into clinically interpretable categories based on observed event rates.

### 2.10. Statistical Analysis

Continuous variables were assessed for normality using the Shapiro–Wilk test. Because the continuous variables did not follow a normal distribution, they were presented as medians with interquartile ranges (IQRs), and between-group comparisons were performed using the Mann–Whitney U test. Categorical variables were presented as counts and percentages and compared using Fisher’s exact test or the chi-squared test, as appropriate. The discriminative performance of the derived scoring system was assessed using receiver operating characteristic analysis and AUC values. For risk stratification, patients were categorized into low-, intermediate-, and high-risk groups according to the total score, considering the observed prevalence and clinical interpretability. The prevalence of complicated appendicitis and the corresponding 95% confidence intervals (CIs) were calculated for each group.

All statistical analyses were performed using R software (version 4.5.2; R Foundation for Statistical Computing, Vienna, Austria). A two-sided *p*-value < 0.05 was considered statistically significant.

## 3. Results

### 3.1. Participant Characteristics

**Figure 1:**
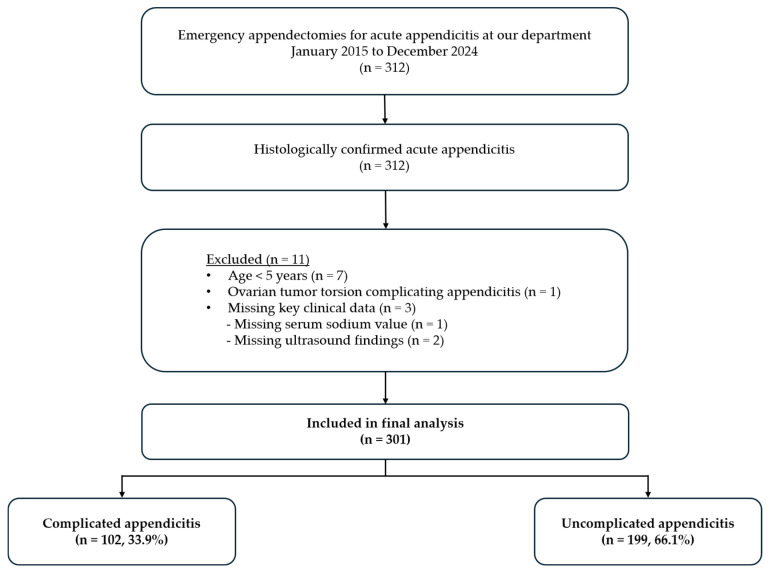
Flow chart of patient selection.

**Figure 2:**
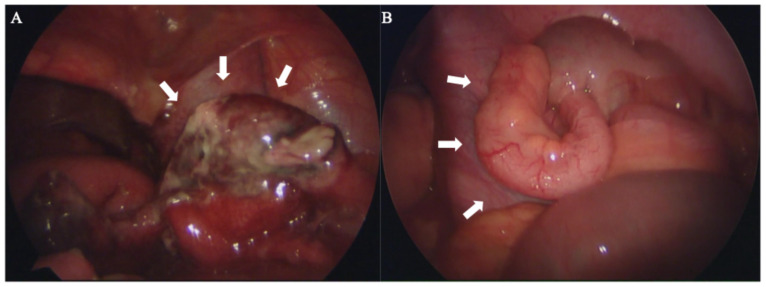
Representative intraoperative findings. (**A**) Complicated appendicitis. (**B**) Uncomplicated appendicitis. White arrows indicate the appendix.

During the study period, 312 emergency appendectomies were performed for acute appendicitis in our department, all of which were histologically confirmed as acute appendicitis. We excluded seven patients younger than 5 years, one patient with ovarian tumor torsion complicating appendicitis, and three patients with missing key clinical data (one lacking a serum sodium value and two lacking ultrasound findings). A total of 301 patients were included in the final analysis; of these, 102 (33.9%) were classified as having complicated appendicitis (complicated group) and 199 (66.1%) as uncomplicated appendicitis (uncomplicated group) (Figure 1). Representative intraoperative findings of complicated and uncomplicated appendicitis are shown in Figure 2.

Patients in the complicated group were significantly younger than those in the uncomplicated group (*p* = 0.049). The median (IQR) body temperature at presentation was higher in the complicated group than in the uncomplicated group (38.0 [37.4–38.5] °C vs. 37.0 [36.7–37.7] °C, *p* < 0.001). Signs of peritoneal irritation were also more frequent in the complicated group (71.6% vs. 52.3%, *p* < 0.001) than in the uncomplicated group. Laboratory findings indicated a more pronounced inflammatory response in the complicated group, with higher WBC counts and CRP levels and lower serum sodium levels than those in the uncomplicated group (all *p* < 0.001). Presence of periappendicular free fluid was significantly more frequent in the complicated group than in the uncomplicated group (40.2% vs. 9.5%, *p* < 0.001; Table 1).

### 3.2. Model Development

In the Firth penalized multivariable logistic regression analysis, body temperature ≥ 38.0 °C (odds ratio [OR] 2.82, 95% CI 1.45–5.51; *p* = 0.002), presence of periappendicular free fluid (OR 3.26, 95% CI 1.50–7.21; *p* = 0.003), serum sodium level < 135 mEq/L (OR 5.37, 95% CI 2.03–16.04; *p* < 0.001), WBC count > 12,000/μL (OR 2.92, 95% CI 1.38–6.55; *p* = 0.005), and CRP level ≥ 3.0 mg/dL (OR 7.11, 95% CI 3.74–13.91; *p* < 0.001) were independently associated with complicated appendicitis (Table 2).

**Figure 3:**
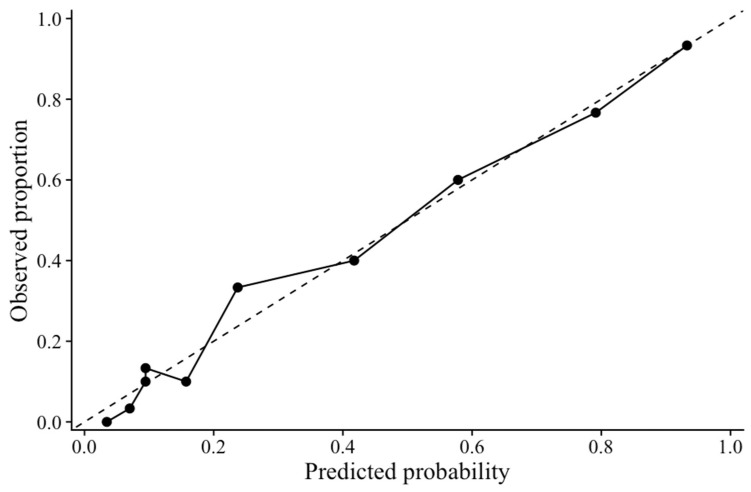
Calibration plot of the multivariable logistic regression model.

Internal validation using bootstrap resampling demonstrated stable discriminative performance of the multivariable logistic regression model. The apparent AUC was 0.882. The mean optimism estimated by bootstrap internal validation was 0.007, resulting in an optimism-corrected AUC of 0.875, indicating minimal overfitting. Calibration analysis demonstrated good agreement between the mean predicted probabilities and observed proportions across the deciles of predicted risk (Figure 3). The Hosmer–Lemeshow goodness-of-fit test indicated no significant lack of fit (*p* = 0.87).

The predictors retained in the final multivariable model were integrated into an additive clinical scoring system. Integer point values were assigned in proportion to the regression coefficients: serum sodium level < 135 mEq/L and CRP level ≥ 3.0 mg/dL were each assigned 2 points, whereas body temperature ≥ 38.0 °C, presence of periappendicular free fluid, and WBC count > 12,000/μL were each assigned 1 point (Table 2). The total score was calculated as the sum of the assigned points, ranging from 0 to 7.

### 3.3. Performance of the Scoring System

**Figure 4:**
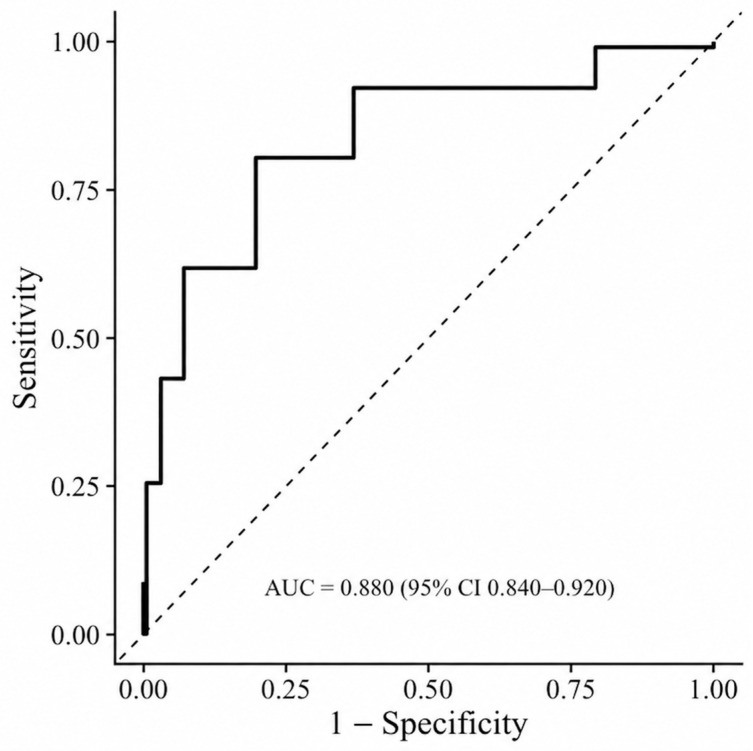
Receiver operating characteristic curve for the total score. The score-based model demonstrates good discriminative ability for predicting complicated appendicitis, with an area under the curve of 0.880 (95% confidence interval, 0.840–0.920).

The scoring system demonstrated good discriminative performance, with an AUC of 0.880 (95% CI, 0.840–0.920) for distinguishing between complicated and uncomplicated appendicitis (Figure 4). The diagnostic performance measures across score thresholds, including sensitivity, specificity, positive predictive value, and negative predictive value, are summarized in Table 3. The risk of complicated appendicitis increased progressively with higher total scores (Table 4). Patients were stratified into three risk categories: low risk (score 0–1), intermediate risk (score 2–4), and high risk (score ≥ 5). Accordingly, the proportion of complicated appendicitis was 6.0% (95% CI, 2.6–11.5) in the low-risk group, 41.4% (95% CI, 32.9–50.3) in the intermediate-risk group, and 88.0% (95% CI, 75.7–95.5) in the high-risk group (Table 4).

## 4. Discussion

In this study, we developed a simple, bedside-applicable scoring system for predicting complicated appendicitis in children aged 5 to <16 years using a small number of routinely available preoperative variables. The score demonstrated good discriminative performance and enabled clear and clinically interpretable risk stratification. By relying on prespecified predictors supported by prior evidence, rather than data-driven optimization within a single cohort, the model emphasizes robustness and practical usability in routine clinical practice. Therefore, this scoring system may serve as a supportive tool for early risk assessment and clinical decision-making in children with acute appendicitis.

In practice, the clinical value of this score lies in its ability to provide clearly defined rule-out and rule-in thresholds. The score may be used after routine laboratory testing and ultrasonography to support three-level clinical decision-making: low-risk patients may be considered for less urgent or nonoperative strategies, high-risk patients may require early surgical prioritization and intensified perioperative planning, and intermediate-risk patients may warrant further diagnostic evaluation. Using a low-risk cut-off of <2 points, the model demonstrated a high sensitivity (0.922) and negative predictive value (0.940), with an observed prevalence of complicated appendicitis of only 6.0% in this group. Clinical guidelines and large-scale studies have reported nonoperative management with antibiotics to be feasible, safe, and effective in uncomplicated appendicitis [17,23,24]. Within this context, a low-risk classification by the present score may facilitate structured shared decision-making regarding optimal treatment selection, including nonoperative management, where such approaches are supported by institutional practice. In contrast, application of a high-risk cut-off of ≥5 points yielded strong rule-in characteristics, with a specificity of 0.970 and a positive predictive value of 0.880, indicating that a high total score substantially increases the likelihood of complicated appendicitis. In such patients, early escalation of surgical and perioperative planning, including timely appendectomy and intensified perioperative management, may be warranted. Preoperative high-risk classification may also help clinicians anticipate the need for broader empirical antibiotic coverage and a potentially longer treatment course. Furthermore, patients classified as having intermediate risk represent a clinically important subgroup. In the present cohort, 38.9% of patients fell into this subgroup. For such patients, further diagnostic evaluation may be warranted, and predictive performance could potentially be improved by integrating additional clinical and imaging findings that have been associated with pediatric complicated appendicitis, such as signs of peritoneal irritation or appendiceal diameter [11,13,19]. However, these assessments are inherently dependent on examiner’s experience and technical proficiency, and this limitation should be carefully considered when applying such approaches in routine clinical practice.

Previously established scoring tools, such as the Alvarado score [25], Pediatric Appendicitis Score (PAS) [26], and Appendicitis Inflammatory Response (AIR) score [27], were developed primarily to support the diagnosis of appendicitis, rather than to predict disease severity. Accordingly, their ability to accurately identify complicated appendicitis in children has been shown to be limited. For example, Lee et al. reported that, in pediatric patients aged < 10 years, the discriminative performance of the commonly used appendicitis scores for identifying complicated appendicitis associated with prolonged hospitalization or readmission was modest, with AUC values of 0.547 for the Alvarado score and 0.571 for the PAS; even the AIR score, which incorporates CRP, achieved only moderate discrimination (AUC = 0.780) [16].

Furthermore, an important practical advantage of the present scoring system is that several of its components overlap with variables that are already familiar to clinicians through these widely used diagnostic scoring systems. Fever and inflammatory markers, such as WBC and CRP, which are core elements of the Alvarado, PAS, and AIR scores, were also incorporated into our model. This overlap may facilitate seamless integration into routine clinical workflows, enabling clinicians to simultaneously assess the likelihood of appendicitis and the risk of complicated disease without adding substantial cognitive or logistical burden. Concurrently, the present score is distinguished by the fact that it is composed exclusively of objective and quantitative variables, without relying on physical examination findings that are inherently subjective. In addition to routinely obtained clinical history and laboratory data, the model incorporates a commonly used ultrasonographic feature—periappendicular free fluid—which directly reflects disease severity, can be relatively easily identified even by less experienced operators, and reduces reliance on subjective physical examination findings. This characteristic is particularly relevant in pediatric patients, in whom physical examination findings may be subtle or variably interpreted [16], and likely contributes to the favorable discriminative performance and risk stratification observed in the present study.

In our prediction model for complicated appendicitis, two predictors—CRP level ≥ 3 mg/dL and serum sodium level < 135 mEq/L—emerged as particularly salient (assigned score 2). Although an elevated CRP level has long been recognized as an established risk factor and predictor of complicated appendicitis, serum sodium has only recently gained attention as a robust prognostic indicator of this condition [7,10,28]. According to a prospective cohort study by Elgendy et al., serum sodium level < 135 mmol/L helped identify perforated or gangrenous appendicitis with a sensitivity and specificity of 94% and 91%, respectively (AUC = 0.981) [7]. The proposed mechanism involves inflammation-induced inappropriate antidiuretic hormone secretion, in which severe infection and cytokine activation—particularly interleukin-6—promote the non-osmotic release of antidiuretic hormone, leading to water retention and dilutional hyponatremia [29]. Moreover, numerous blood-based biomarkers have been reported to demonstrate relatively good discriminative performance in predicting complicated appendicitis [30,31,32]. However, it is reasonable to assume that many of these laboratory variables are highly interrelated and share substantial multicollinearity [33]. Consequently, even if additional biomarkers were incorporated into a prediction model, the incremental gain in discriminative ability would likely be limited, whereas model complexity would increase, potentially compromising bedside usability. In this context, the balance achieved by the present scoring system, which combines strong discriminative performance, clinically interpretable risk stratification, and structural simplicity, is critical for its acceptance and implementation in the real-world initial management of children with acute appendicitis.

This study has some limitations. First, this was a single-center, retrospective observational study of children who underwent emergency appendectomy, which may limit the generalizability of our findings to other institutions with different patient populations, diagnostic pathways, and perioperative management strategies. Additionally, although all eligible patients in this cohort had histopathologically confirmed acute appendicitis, negative appendectomy or alternative non-appendicitis pathology may occur in broader real-world populations of children evaluated for suspected appendicitis. Therefore, further validation in such broader suspected-appendicitis cohorts is warranted before the proposed scoring system is applied beyond children with confirmed acute appendicitis. Second, this study included only patients who underwent emergency surgery after being diagnosed with acute appendicitis, because emergency surgery was the standard management strategy at our institution during the study period. Therefore, patients who may have been initially misdiagnosed with other conditions, such as gastroenteritis, and were managed nonoperatively by other departments were not included. Third, because our institution is a tertiary referral center, most patients were referred from other hospitals. Therefore, pre-referral or pre-sampling intravenous fluid administration may have influenced laboratory values, including serum sodium levels. Fourth, ultrasound findings were based on routine clinical assessments, and inter-operator variability may have affected the evaluation of periappendicular free fluid. Finally, although internal validation demonstrated minimal optimism and acceptable calibration, external validation is required to confirm the generalizability and robustness of the proposed scoring system. Future validation should be performed in independent, preferably multicenter, cohorts. These cohorts should reflect differences in patient populations, referral patterns, ultrasound experience, and management strategies. Future studies should assess discrimination, calibration, and patient distribution across the proposed risk groups. Potential implementation challenges include variation in blood-sampling timing, pre-referral fluid administration, and the reproducibility of ultrasound-based assessment of periappendicular free fluid.

## 5. Conclusions

We developed a simple, additive scoring system for predicting complicated appendicitis in children aged 5 to <16 years using a small set of routinely available preoperative variables. The scoring system demonstrated good discriminative performance and enabled clinically interpretable risk stratification while emphasizing robustness and bedside applicability using prespecified predictors. Although external validation is required, this pragmatic scoring system may support standardized early risk assessment and clinical decision-making in cases of pediatric acute appendicitis. In particular, early identification of high-risk patients may help facilitate timely surgical prioritization and appropriate perioperative antibiotic management, potentially contributing to therapeutic advantages in clinical practice.

## Figures and Tables

**Table 1:** Comparison of patient characteristics between the complicated and uncomplicated groups.

	Complicated Group	Uncomplicated Group	*p*-Value
	n = 102	n = 199	
Patient characteristic			
Male sex, n (%)	70 (68.6)	119 (59.8)	0.20
Age (y), median (IQR)	10.0 (8.0–12.0)	11.0 (9.0–13.0)	0.049
Height (cm), median (IQR)	140.0 (127.3–150.0)	142.0 (132.0–152.8)	0.14
Body weight (kg), median (IQR)	32.4 (25.9–42.9)	36.0 (27.3–45.0)	0.20
BMI (kg/m^2^), median (IQR)	16.9 (15.4–19.4)	17.2 (15.7–19.6)	0.40
Body temperature (°C), median (IQR)	38.0 (37.4–38.5)	37.0 (36.7–37.7)	<0.001
Peritoneal irritation signs, n (%)	73 (71.6)	104 (52.3)	<0.001
Laboratory finding			
WBC count (/μL), median (IQR)	16,000 (13,100–19,700)	14,000 (10,600–16,900)	<0.001
Hemoglobin level (g/dL), median (IQR)	13.1 (12.5–14.0)	12.9 (12.4–13.7)	0.20
Platelet count (×10^9^/L), median (IQR)	286 (237–330)	268 (234–311)	0.10
Sodium level (mEq/L), median (IQR)	136 (134–138)	138 (137–140)	<0.001
CRP level (mg/dL), median (IQR)	6.3 (3.6–13.5)	1.4 (0.4–3.0)	<0.001
Ultrasound finding			
Periappendicular free fluid, n (%)	41 (40.2)	19 (9.5)	<0.001
Components of complicated appendicitis ^a^			
Gangrene, n (%)	96 (94.1)	-	
Perforation, n (%)	40 (39.2)	-	
Abscess formation, n (%)	31 (30.4)	-	

^a^ Not mutually exclusive; WBC: white blood cell; CRP: C-reactive protein; BMI: body mass index; IQR: interquartile range.

**Table 2:** Firth penalized multivariable logistic regression analysis for predictors of complicated appendicitis.

Variable	Regression Coefficient	Odds Ratio	95% CI	*p*-Value	Assigned Score
Body temperature ≥ 38.0 °C	1.04	2.82	1.45–5.51	0.002	1
Periappendicular free fluid	1.18	3.26	1.50–7.21	0.003	1
Sodium level < 135 mEq/L	1.68	5.37	2.03–16.04	<0.001	2
WBC count > 12,000/μL	1.07	2.92	1.38–6.55	0.005	1
CRP level ≥ 3.0 mg/dL	1.96	7.11	3.74–13.91	<0.001	2

CI: confidence interval; WBC: white blood cell; CRP: C–reactive protein.

**Table 3:** Predictive performance of the scoring system for complicated appendicitis at each score threshold.

Cutoff Score	Sensitivity	Specificity	PPV	NPV
≥0	1.000	0.000	0.340	NA
≥1	0.990	0.207	0.391	0.976
≥2	0.922	0.631	0.563	0.940
≥3	0.804	0.803	0.678	0.888
≥4	0.618	0.929	0.818	0.825
≥5	0.431	0.970	0.880	0.768
≥6	0.255	0.995	0.963	0.722
≥7	0.088	1.000	1.000	0.680

NA: not applicable; PPV: positive predictive value; NPV: negative predictive value.

**Table 4:** Prevalence of complicated appendicitis according to total score and risk category.

Score	n	Complicated Appendicitis, n (%)	Risk Category	Prevalence of Complicated Appendicitis, % (95% CI)
0	42	1 (2.4)	Low	6.0 (2.6–11.5)
1	91	7 (7.7)		
2	46	12 (26.1)	Intermediate	41.4 (32.9–50.3)
3	44	19 (43.2)		
4	27	19 (70.4)		
5	23	18 (78.3)	High	88.0 (75.7–95.5)
6	18	17 (94.4)		
7	9	9 (100)		

CI: confidence interval.

## Data Availability

The data presented in this study are available on request from the corresponding author upon reasonable request.

## Abbreviations

The following abbreviations are used in this manuscript:

AIR	Appendicitis Inflammatory Response
AUC	area under the ROC curve
CI	confidence interval
CRP	C-reactive protein
IQR	interquartile range
OR	odds ratio
PAS	Pediatric Appendicitis Score
WBC	white blood cell
